# Association of healthy sleep patterns with incident cervical disc disorders: a prospective study

**DOI:** 10.3389/fnins.2026.1940620

**Published:** 2026-09-28

**Authors:** Wanyue Li, Yanzhao Hao, Ruihua Sun, Xiguo Cai, Zhiqing Chen, Shuxiao Ma, Weisheng Zhuang

**Affiliations:** 1 Henan Provincial People's Hospital & People's Hospital of Zhengzhou University, Zhengzhou, China; 2 Department of Rehabilitation, Henan Provincial People's Hospital & People's Hospital of Zhengzhou University, Zhengzhou, China; 3 Department of Neurology, Beijing Tiantan Hospital, Capital Medical University, Beijing, China

**Keywords:** cervical disc disorders, cohort study, interaction effect, sleep pattern, UK Biobank

## Abstract

**Background:**

There is limited evidence regarding the relationship between multidimensional sleep behaviors and incident cervical disc disorders. This study examined whether a composite healthy sleep pattern was associated with the subsequent risk of cervical disc disorders.

**Methods:**

This prospective cohort study used data from the UK Biobank. Participants with baseline cervical disc disorders or missing data were excluded, leaving 399,862 participants for analysis. A composite healthy sleep score was derived from five sleep characteristics: sleep chronotype, sleep duration, insomnia, snoring, and daytime sleepiness. The primary outcome was incident cervical disc disorders, identified using ICD-10 codes (M50). Cox proportional hazards models were fitted with adjustment for demographic, lifestyle, and socioeconomic covariates.

**Results:**

During follow-up, 2,735 incident cases were documented. Each 1-point increase in the healthy sleep score was associated with an 18% lower risk (HR = 0.82; 95% CI: 0.79–0.85). Participants with a score of 5 had a lower risk than those with a score of 0–1 (HR = 0.43; 95% CI: 0.35–0.52). The inverse association was stronger among participants with higher socioeconomic deprivation (*P for interaction* = 0.018).

**Conclusion:**

In this cohort analysis, baseline adherence to healthy sleep behaviors was associated with a significantly lower risk of cervical disc disorders, suggesting that promoting healthy sleep may be a viable strategy for primary prevention.

## 1 Introduction

Cervical disc disorders are among the most common degenerative conditions of the cervical spine and a leading cause of neck pain and functional impairment worldwide, though neck pain has multiple etiologies (GBD 2021 Neck Pain Collaborators, 2024). Their prevalence has risen markedly with population aging and lifestyle changes (GBD 2021 Neck Pain Collaborators, 2024; Cieza et al., 2021; Kazeminasab et al., 2022). Cervical disc disorders are rarely cured definitively, substantially reducing patients’ work capacity and quality of life (Kazeminasab et al., 2022). Identifying modifiable risk factors is therefore essential for developing prevention strategies. Sleep health has emerged as a key factor in maintaining overall health and preventing chronic disease (Institute of Medicine Committee on Sleep, Medicine, and Research, 2006; Chaput et al., 2020). Sleep is a multidimensional physiological process involving duration, quality, rhythm, and alertness (Zou et al., 2025). In musculoskeletal disorders, cross-sectional studies have reported consistent associations between poor sleep and increased pain (Afolalu et al., 2018; Sha et al., 2025). Prospective studies are fewer, and most have examined single sleep dimensions rather than multidimensional constructs. Moreover, most focused on existing pain rather than incident disease. Sleep deprivation lowers pain thresholds and enhances central sensitization (Krause et al., 2019), but whether these mechanisms translate to clinical disease onset remains unclear.

Several biological pathways may link sleep to cervical disc disorders. Disc cells contain intrinsic circadian clocks that regulate metabolism and matrix synthesis (Zhao and Wang, 2026). Circadian disruption increases inflammation and impairs nutrient supply (Zhao and Wang, 2026). Lower melatonin levels in disc degeneration patients are associated with elevated IL-6 and TNF-α (Tian et al., 2021). Sleep fragmentation also increases central sensitization, amplifying minor tissue changes into clinical symptoms (Chang et al., 2022).

Despite the recognized association between sleep and pain, studies examining sleep patterns as determinants of cervical disc disorders remain limited (Hurwitz et al., 2018). Three major limitations exist. First, most studies used single sleep indicators, which cannot capture the multidimensional nature of sleep (Huang et al., 2025; Gustafsson et al., 2017). Composite scores provide more comprehensive exposure assessment (Li et al., 2021). Second, sample sizes were often small, limiting statistical power (Kolenkiewicz et al., 2018). Third, many studies were cross-sectional or had short follow-up, limiting temporal assessment. Large-scale prospective evidence linking multidimensional sleep patterns to incident cervical disc disorders is critically lacking.

Previous studies on sleep and musculoskeletal health fall into two categories. Small-scale studies used detailed objective assessments like polysomnography (Stroemel-Scheder et al., 2019; Abeler et al., 2020) but had limited statistical power. Large-scale epidemiological studies had adequate power but used simplified sleep measures that could not capture multidimensional sleep (Van Looveren et al., 2021). Our study integrates both strengths: a large population-based sample, a multidimensional sleep score, and hospital-recorded ICD-10 outcomes. This combination has not been available in previous studies.

Using data from the UK Biobank, we investigated the association between a multidimensional healthy sleep pattern and the subsequent risk of cervical disc disorders. The healthy sleep pattern incorporated five domains: chronotype, duration, insomnia, snoring, and daytime sleepiness. Our study has three novel contributions. Methodologically, we construct a composite sleep score capturing five dimensions. Epidemiologically, we focus on incident disease rather than pain symptoms. Practically, we combine a large sample, prospective design, and long follow-up. This allows us to examine the temporal relationship with greater statistical power and precision.

## 2 Methods

### 2.1 Study design

This study utilized data from the UKB, a cohort that successfully recruited over 500,000 general adults aged 40 to 70 between 2006 and 2010. A multidimensional data collection strategy was employed for the baseline assessment, which included a standardized touch-screen questionnaire, professionally conducted face-to-face in-depth interviews, and systematic documentation of demographic characteristics, life course exposures, and medical history. All participants underwent a standardized physical examination and provided high-quality biological samples. Following this, the research team conducted ongoing long-term longitudinal follow-up and updates of these key indicators (Yévenes-Briones et al., 2022; Sudlow et al., 2015).

UKB has ethical approval from the North West Multi-centre Research Ethics Committee (MREC) as a Research Tissue Bank (RTB). All participants provided written informed consent at recruitment. This study is a secondary analysis of de-identified UKB data. No separate ethical approval was required for this analysis, as it was conducted under the UKB RTB approval framework. All participants provided written informed consent (Sudlow et al., 2015). During the data analysis phase, we excluded individuals who did not meet the inclusion criteria: those with missing data on all dimensions of the sleep score (*n* = 91,705), those with missing covariates (*n* = 8,880), and participants with pre-existing cervical disc disorders at baseline (*n* = 1,489). The final effective sample size for statistical analysis comprised 399,862 participants (see Figure 1 for details).

**Figure 1:**
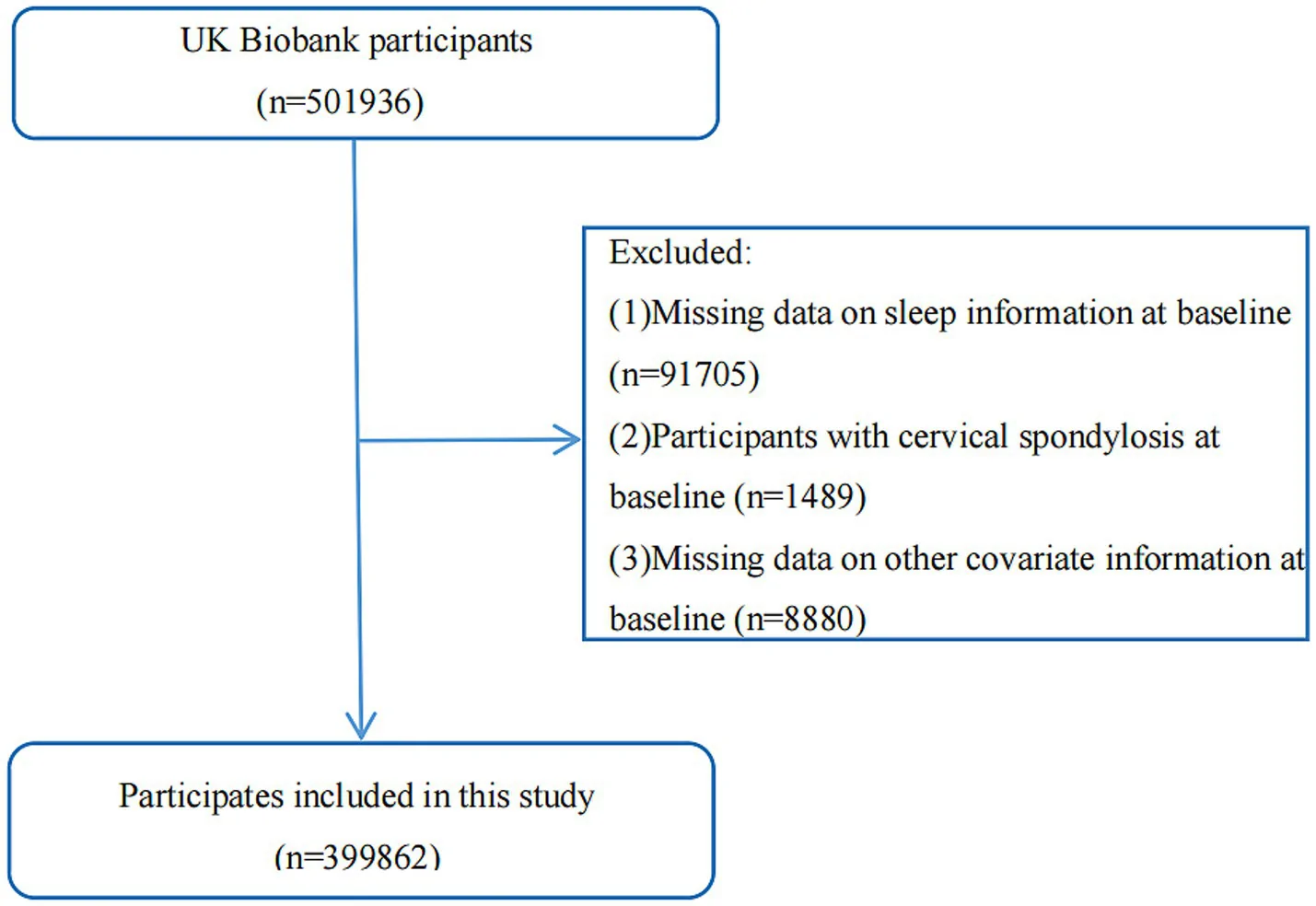
Flowchart for the participants who were included in the analysis.

### 2.2 Ascertainment of exposure

Data on various sleep behaviors, including sleep chronotype, sleep duration, daytime sleepiness, insomnia, and snoring, were collected at baseline (see Supplementary Table 1 for detailed questionnaire content). In this study, healthy sleep patterns were defined as a combination of behaviors encompassing five dimensions. These behaviors were classified as “low risk” and “high risk” according to established criteria in the literature. Healthy sleep behaviours were defined as follows: an early chronotype (“morning” person or “morning” person rather than an “evening” person); a sleep duration of 7–8 h per day; no frequent insomnia symptoms (“sometimes” or “never/rarely”); no self-reported snoring; and no frequent daytime sleepiness (“sometimes” or “never/rarely”). A score of 1 was assigned to each of these criteria, while a score of 0 was given to all other criteria. The final composite healthy sleep score, which ranges from 0 to 5, is the sum of the scores for each indicator, with higher scores indicating a healthier sleep pattern (Zou et al., 2025; Shi et al., 2022; Fan et al., 2020). It is important to note that all sleep behaviors were assessed at baseline only. We did not collect repeated measurements during follow-up. Therefore, our exposure definition reflects sleep status at study entry rather than long-term sleep patterns. For the composite sleep score, sleep duration was dichotomized as 7–8 h versus other durations. This binary classification was used for score calculation. For the separate analysis of individual sleep behaviors (presented in Supplementary Table 4), sleep duration was categorized into three groups: <7 h, 7–8 h, and >9 h. This allowed examination of both short and long sleep durations as separate risk categories.

### 2.3 Ascertainment of outcome

The primary outcome was incident cervical disc disorders. The outcome was operationally defined using an ICD-10-based phenotype derived from UKB hospital inpatient records. Specifically, we used Field 41,270 (Diagnoses - ICD10), which contains summary diagnosis records from hospital inpatient admissions (Flachs et al., 2022). Participants were classified as having a cervical disc disorder if any of the following ICD-10 codes was recorded after baseline: M50.0, cervical disc disorder with myelopathy; M50.1, cervical disc disorder with radiculopathy; M50.2, other cervical disc displacement; M50.3, other cervical disc degeneration; M50.8, other cervical disc disorders; or M50.9, cervical disc disorder, unspecified (Flachs et al., 2022; Shlykov et al., 2023; Kolenkiewicz et al., 2018). It is important to note that ICD-10 code M50 specifically represents cervical disc disorders, while code M47 represents spondylosis. These are distinct but related categories in the ICD-10 classification. We selected M50 codes for our primary outcome because they cover the most clinically significant forms of cervical degenerative disease requiring hospital admission, including disc displacement, degeneration, and disorders with myelopathy or radiculopathy. This approach is consistent with established UKB phenotype definitions for cervical disc disorders.

The first recorded diagnosis after study enrollment was considered the incident event. The corresponding diagnosis date was obtained from the UKB inpatient diagnosis date field (Field 41,280), which records the first date on which each ICD-10 diagnosis was documented in the hospital inpatient data. Participants were followed from enrollment until the first occurrence of the outcome, death, loss to follow-up, or August 31, 2025, whichever occurred first. Because the M50 category represents cervical disc disorders rather than the full clinical spectrum of cervical spondylosis, the present study uses “cervical disc disorders” to describe the ICD-10-defined outcome throughout the manuscript. In this study, “cervical disc disorders” refers specifically to the ICD-10-defined M50 phenotype and should not be interpreted as synonymous with the broader clinical diagnosis of cervical spondylosis.

### 2.4 Ascertainment of covariates

To mitigate the effects of specific confounders, we considered several baseline covariates: age, sex (male or female), ethnicity (white or other), education level (university/college degree or other), Townsend Deprivation Index (TDI), household income, body mass index (BMI), smoking status (never, previous, or current), alcohol status (never, previous, or current), vascular/heart problems, and metabolic equivalent (MET) time per week (“Summed MET minutes per week for all activity”). The Townsend Deprivation Index is a composite measure of area-level socioeconomic status. It is derived from national census data. Higher scores indicate greater material deprivation. Lower scores indicate less deprivation. Negative values reflect relative affluence. Covariates were selected *a priori* based on a prespecified causal framework informed by previous literature. Variables were considered as potential confounders if they met two criteria: (1) they were independently associated with cervical disc disorders in prior observational studies, and (2) they were also associated with sleep patterns. Demographic confounders included age, sex, and ethnicity. These factors are well established as correlates of both sleep health and musculoskeletal outcomes. Socioeconomic confounders included education, household income, and the Townsend Deprivation Index. These factors influence both sleep quality and healthcare access. Lifestyle confounders included BMI, smoking status, alcohol consumption, and physical activity (MET time per week). These factors are known to affect both sleep and degenerative joint disease. Vascular or heart problems were also included as a comorbidity indicator. We did not adjust for potential mediators, such as pain symptoms or analgesic use. This was to avoid overadjustment bias. All covariates were measured at baseline using standardized UKB protocols.

### 2.5 Statistical analyses

Missing data for covariates (household income and MET time per week) were imputed using multivariate imputation by chained equations (Shah et al., 2014). Depending on the distribution of healthy sleep scores, mean values or percentages were used to characterize participants at baseline. Given the small number of individuals with scores of 0 and 1 in the healthy sleep score, we combined them into a single category for analysis. To assess the association between the healthy sleep score and the risk of incident cervical disc disorders, we constructed Cox proportional hazards regression models. The model considered the score both as an ordered categorical variable and as a continuous variable (each additional point). Ultimately, in addition to the unadjusted model, we fitted two corrected models: model 1 controlled for age, sex, ethnicity, household income, educational level, and TDI; model 2 further adjusted for BMI, smoking status, alcohol status, have or no Vascular/heart problems, and MET time per week. Subgroup analyses were performed based on age (< 60, ≥ 60 years), sex (male, female), TDI (< 0, ≥ 0), BMI (< 30, ≥ 30 kg/m^2^), educational level (College/University, Other), and household income (< £52,000, ≥ £52,000) to identify potential effects of change. The interaction between each stratification factor and the healthy sleep score in relation to the risk of cervical disc disorders and the healthy sleep score was tested via the likelihood ratio test, which compared models both with and without a cross-product term.

In this study, we conducted a series of sensitivity analyses to assess the robustness of our findings. First, to eliminate the possibility of reverse causation, we excluded individuals who developed cervical disc disorders within 3 or 5 years of follow-up. Second, to account for demographic confounders, we retained only white participants and excluded individuals from other racial groups. Third, we censored follow-up data on March 11, 2020, the date when COVID-19 was declared a pandemic by the World Health Organization. Follow-up time after this date was excluded to avoid potential pandemic-related influences on health behaviors and outcome ascertainment. Fourth, we computed weighted healthy-sleep scores from five sleep behaviors, assigning weights according to adjusted hazard ratios (HRs) estimated by the Cox proportional hazards model. Fifth, we added baseline ICD-10 diagnoses of M51 (other intervertebral disc disorders) and M54 (dorsalgia, including neck pain) as binary covariates to Model 2 and re-ran all analyses. Sixth, we excluded all participants with a baseline diagnosis of M51 or M54 and re-analyzed the primary Cox model within this restricted cohort. Seventh, we performed an additional covariate adjustment sensitivity analysis. We added dorsalgia, depression, and sitting time (hours/day) as extra covariates to Model 2. The primary Cox model was then re-estimated with these additional adjustments. The proportional hazards assumption was assessed using scaled Schoenfeld residuals. The global test and covariate-specific tests were examined. Evidence of non-proportionality was observed for the healthy sleep score, age, TDI, and BMI. To account for these time-dependent effects, we performed a piecewise Cox regression analysis. Follow-up was divided into two periods (≤10 years and >10 years). The baseline hazard was stratified by follow-up period. Interaction terms between follow-up period and the covariates showing evidence of non-proportionality were included. The modified model was subsequently reassessed using scaled Schoenfeld residuals. All statistical analyses were performed using the R package (version 4.4.2), where *p* values < 0.05 were considered statistically significant.

## 3 Results

### 3.1 Baseline characteristics

The mean follow-up duration was 15.83 years (SD 2.67). The mean age of the included population was 56.45 years (SD 8.09), and 220,309 participants were female (55.10%). The baseline distribution of sleep scores was: 9,641 (2.41%) with 0–1 points, 44,927 (11.24%) with 2 points, 112,450 (28.12%) with 3 points, 147,158 (36.80%) with 4 points, and 85,686 (21.43%) with 5 points. Participants with higher healthy sleep scores had longer activity duration, a lower prevalence of vascular or heart disease, a lower prevalence of smoking, and lower BMI and TDI. Participants with higher healthy sleep scores had lower TDI values, indicating that they lived in more affluent areas (Table 1).

**Table 1:** Baseline characteristics according to healthy sleep score in the cohort.

Characteristics	Overall	Healthy sleep score					*p* value
		0–1	2	3	4	5	
	(*n* = 399,862)	(*n* = 9,641)	(*n* = 44,927)	(*n* = 112,450)	(*n* = 147,158)	(*n* = 85,686)	
Age	56.45 (8.09)	56.58 (7.73)	56.71 (7.82)	56.69 (7.97)	56.37 (8.15)	56.13 (8.31)	<0.001
Sex							
Female	220,309 (55.10)	4,937 (51.21)	23,645 (52.63)	58,125 (51.69)	79,541 (54.05)	54,061 (63.09)	<0.001
Male	179,553 (44.90)	4,704 (48.79)	21,282 (47.37)	54,325 (48.31)	67,617 (45.95)	31,625 (36.91)	
BMI (kg/m^2^)	27.38 (4.76)	30.05 (5.87)	28.71 (5.27)	27.86 (4.83)	27.11 (4.53)	26.23 (4.26)	<0.001
Townsend deprivation index	−1.43 (3.02)	−0.62 (3.36)	−1.07 (3.19)	−1.34 (3.06)	−1.53 (2.95)	−1.64 (2.90)	<0.001
MET time/week(h)	44.12 (43.97)	39.60 (44.66)	41.32 (44.07)	43.07 (43.88)	44.55 (43.73)	46.74 (44.20)	<0.001
Ethnicity							
Mixed people	2,274 (0.57)	79 (0.82)	315 (0.70)	656 (0.58)	802 (0.54)	422 (0.49)	<0.001
Other people	16,842 (4.21)	571 (5.92)	2036 (4.53)	4,815 (4.28)	6,068 (4.12)	3,352 (3.91)	
White people	380,746 (95.22)	8,991 (93.26)	42,576 (94.77)	106,979 (95.13)	140,288 (95.33)	81,912 (95.60)	
Education							
College/University	132,218 (33.07)	2,371 (24.59)	12,412 (27.63)	34,902 (31.04)	50,933 (34.61)	31,600 (36.88)	<0.001
Other	267,644 (66.93)	7,270 (75.41)	32,515 (72.37)	77,548 (68.96)	96,225 (65.39)	54,086 (63.12)	
Smoking status							
Current	41,286 (10.33)	1715 (17.79)	6,618 (14.73)	13,459 (11.97)	13,802 (9.38)	5,692 (6.64)	<0.001
Never	218,256 (54.58)	4,137 (42.91)	21,012 (46.77)	57,645 (51.26)	82,141 (55.82)	53,321 (62.23)	
Previous	140,320 (35.09)	3,789 (39.30)	17,297 (38.50)	41,346 (36.77)	51,215 (34.80)	26,673 (31.13)	
Alcohol status							
Current	369,996 (92.53)	8,693 (90.17)	41,341 (92.02)	104,255 (92.71)	136,723 (92.91)	78,984 (92.18)	<0.001
Never	16,276 (4.07)	418 (4.34)	1726 (3.84)	4,354 (3.87)	5,775 (3.92)	4,003 (4.67)	
Previous	13,590 (3.40)	530 (5.50)	1860 (4.14)	3,841 (3.42)	4,660 (3.17)	2,699 (3.15)	
Vascular/heart problems							
No	282,688 (70.70)	5,418 (56.20)	28,590 (63.64)	76,428 (67.97)	106,714 (72.52)	65,538 (76.49)	<0.001
Yes	117,174 (29.30)	4,223 (43.80)	16,337 (36.36)	36,022 (32.03)	40,444 (27.48)	20,148 (23.51)	
Household income							
Less than 18,000	84,278 (21.08)	2,945 (30.55)	11,443 (25.47)	24,707 (21.97)	28,816 (19.58)	16,367 (19.10)	<0.001
18,000 to 30,999	100,385 (25.10)	2,454 (25.45)	11,537 (25.68)	28,685 (25.51)	36,541 (24.83)	21,168 (24.70)	
31,000 to 51,999	105,631 (26.42)	2,231 (23.14)	11,381 (25.33)	29,564 (26.29)	39,626 (26.93)	22,829 (26.64)	
52,000 to 100,000	86,824 (21.71)	1,664 (17.26)	8,602 (19.15)	23,516 (20.91)	33,302 (22.63)	19,740 (23.04)	
Greater than 100,000	22,744 (5.69)	347 (3.60)	1964 (4.37)	5,978 (5.32)	8,873 (6.03)	5,582 (6.51)	
Chronotype							
Evening person	149,145 (37.30)	8,780 (91.07)	32,126 (71.51)	59,318 (52.75)	48,921 (33.24)	–	<0.001
Morning person	250,717 (62.70)	861 (8.93)	12,801 (28.49)	53,132 (47.25)	98,237 (66.76)	–	
Sleep duration 7–8 h/day	126,568 (31.65)	9,222 (95.65)	35,677 (79.41)	55,273 (49.15)	26,396 (17.94)	–	<0.001
Other	273,294 (68.35)	419 (4.35)	9,250 (20.59)	57,177 (50.85)	120,762 (82.06)	–	
Frequent insomnia							
Yes	110,926 (27.74)	9,172 (95.14)	32,545 (72.44)	47,622 (42.35)	21,587 (14.67)	–	<0.001
No	288,936 (72.26)	469 (4.86)	12,382 (27.56)	64,828 (57.65)	125,571 (85.33)	–	
Self-reported snoring							
Yes	148,645 (37.17)	9,021 (93.57)	30,777 (68.50)	59,614 (53.01)	49,233 (33.46)	–	<0.001
No	251,217 (62.83)	620 (6.43)	14,150 (31.50)	52,836 (46.99)	97,925 (66.54)	–	
Frequent daytime sleepiness							
Yes	10,783 (2.70)	3,033 (31.46)	3,656 (8.14)	3,073 (2.73)	1,021 (0.69)	–	<0.001
No	389,079 (97.30)	6,608 (68.54)	41,271 (91.86)	109,377 (97.27)	146,137 (99.31)	–	
Cervical disc disorders							
No	397,127 (99.32)	9,501 (98.55)	44,467 (98.98)	111,615 (99.26)	146,272 (99.40)	–	<0.001
Yes	2,735 (0.68)	140 (1.45)	460 (1.02)	835 (0.74)	886 (0.60)	–	
Year	15.83 (2.67)	15.44 (3.23)	15.63 (2.96)	15.77 (2.75)	15.88 (2.59)	15.96 (2.45)	<0.001

The values for continuous variables are given as mean ± SD and values for categorical variables are given as numbers (percentage).

(−): all participants in this category met the healthy definition for that specific behavior.

Sleep behaviors were measured at baseline and do not reflect changes during follow-up.

BMI, body mass index; MET, metabolic equivalent.

### 3.2 Association between healthy sleep score and incident cervical disc disorders

Over a mean follow-up of 15.83 years (SD 2.67), 2,735 incident cervical disc disorder events were recorded. The crude model indicated a significant inverse association between healthy sleep score and cervical disc disorders risk (*P* trend < 0.001). The inverse association remained after additional covariate adjustment. The risk declined progressively with increasing healthy sleep scores (*P* trend < 0.001). In fully adjusted Model 2, participants with a healthy sleep score of 5 had a substantially lower risk than those with a score of 0–1 (HR, 0.43; 95% CI 0.35–0.52; *P* trend < 0.001). Moreover, each 1-point increase in the healthy sleep score was associated with an 18% lower risk of incident cervical disc disorders (HR, 0.82; 95% CI 0.79–0.85; *P* trend < 0.001) (Table 2).

**Table 2:** Association between healthy sleep score and risk of incident cervical disc disorders.

Characteristics	Healthy sleep score					Per1-point increment	*P* for trend
	0–1	2	3	4	5		
Participants, *n*	9,641	44,927	112,450	147,158	85,686	**–**	**–**
Cervical disc disorders, *n*	140	460	835	886	414	**–**	**–**
Follow-up period, year							
Means ± SD	15.4 (3.23)	15.6 (2.96)	15.8 (2.75)	15.9 (2.59)	16.0 (2.45)	**–**	**–**
Median (IQR)	16.3 [0.0575, 18.4]	16.4 [0.00274, 18.4]	16.4 [0.0110, 18.4]	16.5 [0.0110, 18.4]	16.5 [0.0301, 18.7]	**–**	**–**
Hazard ratio for incident cervical disc disorders (95% CI)							
Crude model	Reference	0.70 (0.58–0.84)	0.50 (0.42–0.60)	0.40 (0.34–0.48)	0.32 (0.27–0.39)	0.77 (0.74–0.79)	<0.001
Adjusted model 1	Reference	0.73 (0.60–0.88)	0.54 (0.45–0.65)	0.45 (0.38–0.54)	0.37 (0.30–0.45)	0.79 (0.76–0.82)	<0.001
Adjusted model 2	Reference	0.78 (0.63–0.93)	0.59(0.49–0.71)	0.50(0.42–0.60)	0.43(0.35–0.52)	0.82 (0.79–0.85)	<0.001

A Cox proportional hazard model was conducted. Adjusted model 1, age, sex, ethnicity, household income, educational level, and Townsend deprivation index were adjusted for in this model; adjusted model 2, body mass index, smoking status, alcohol status, have or no Vascular/heart problems, and MET time per week additionally were adjusted for in this model.

Sleep behaviors were measured at baseline and do not reflect changes during follow-up.

CI, confidence interval.

The proportional hazards assumption was formally assessed using scaled Schoenfeld residuals. In the initial Cox model, significant non-proportionality was detected for the healthy sleep score (*p* = 0.0039), age (*p* < 0.001), TDI (*p* = 0.0083), and BMI (*p* = 0.0058). The global test was also significant (*p* = 1.73 × 10^−5^) (Supplementary Table 3).

We therefore performed a piecewise Cox proportional hazards analysis. Follow-up was divided into two periods (≤10 years and >10 years), and the baseline hazard was stratified by follow-up period. Period-specific interaction terms were included for covariates showing evidence of non-proportionality. The association between the healthy sleep score and incident cervical disc disorders remained significant in both periods. The HR was 0.789 (95% CI, 0.755–0.823; *p* < 0.001) during the first 10 years and 0.887 (95% CI, 0.830–0.947; *p* < 0.001) thereafter. A significant interaction between healthy sleep score and follow-up period was observed (HR, 1.124; 95% CI, 1.040–1.216; *p* = 0.0034) (Supplementary Table 2). The global Schoenfeld test after model modification showed no significant residual violation of the proportional hazards assumption (*p* = 0.125) (Supplementary Table 3).

When individual sleep behaviors were examined separately, an early chronotype, 7–8 h of sleep per day, never/rare insomnia, and absence of frequent daytime sleepiness were associated with 10, 29, 34, and 32% lower risks of incident cervical disc disorders, respectively (Figure 2). Supplementary Table 4 presents the detailed associations for individual sleep behaviors. After multivariable adjustment, the associations for snoring and evening chronotype were no longer statistically significant. When individual sleep behaviors were analyzed separately (using three-category classification for sleep duration, as described in Methods 2.2), an early chronotype, 7–8 h of sleep per day, never/rare insomnia, and absence of frequent daytime sleepiness were associated with 10, 29, 34, and 32% lower risks of incident cervical disc disorders, respectively (Figure 2). Supplementary Table 4 presents the full results for individual sleep behaviors. In the fully adjusted model, the associations for snoring and evening chronotype were no longer statistically significant (Supplementary Table 4). This suggests that their apparent effects may be partly explained by other covariates.

**Figure 2:**
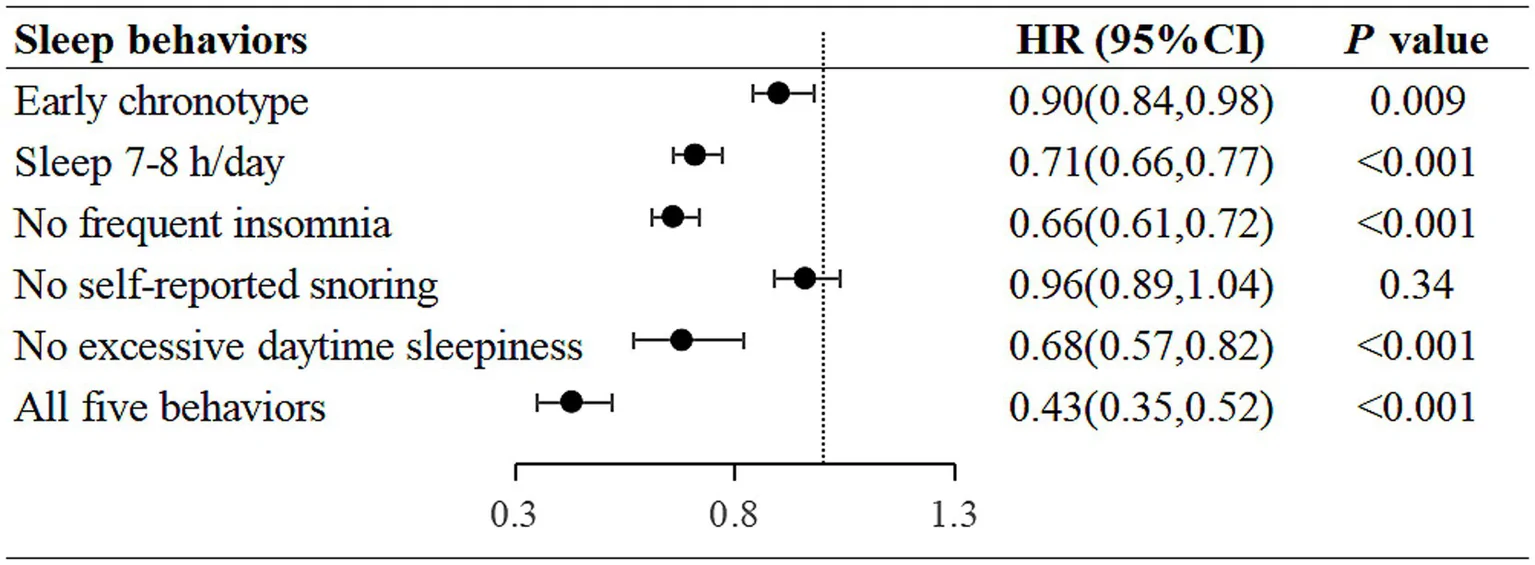
Associations of individual healthy sleep behaviours with the risk of incident cervical disc disorders.

### 3.3 Subgroup analyses

We conducted subgroup analyses by sex, age, and TDI to identify potential moderators; only TDI showed a significant interaction. The inverse association was stronger among participants with TDI ≥ 0 (greater socioeconomic deprivation) than among those with TDI < 0 (less deprivation) (*P* interaction < 0.05) (Table 3). Comparing a healthy sleep score of 5 with a score of 0–1, the corresponding HRs for incident cervical disc disorders were 0.46 (95% CI, 0.36–0.60) among participants with TDI < 0 and 0.34 (95% CI, 0.25–0.46) among those with TDI ≥ 0 (Table 3).

**Table 3:** Associations between healthy sleep score and the risk of incident cervical disc disorders across prespecified subgroups.

Subgroups	Adjusted hazard ratio* (95%CI)					*P* for trend	*P* for interaction
	Healthy sleep score						
	0–1	2	3	4	5		
Age							0.156
<60 years (*n* = 228,372)	1 (reference)	0.75 (0.59–0.96)	0.52 (0.41–0.66)	0.48 (0.38–0. 60)	0.39 (0.30–0. 51)	<0.001	
≥60 years (*n* = 171,490)	1 (reference)	0.81 (0.60–1. 11)	0.71 (0.53–0. 95)	0.56 (0.42–0. 75)	0.49 (0.36–0. 67)	<0.001	
Sex							0.664
Male (*n* = 179,553)	1 (reference)	0.77(0.59–1.00)	0.56 (0.43–0. 72)	0.47 (0.36–0. 60)	0.43 (0.32–0. 56)	<0.001	
Female (*n* = 220,309)	1 (reference)	0.77 (0.59–1. 01)	0.63 (0.48–0.81)	0.54 (0.42–0.70)	0.43 (0.33–0. 57)	<0.001	
Townsend deprivation index							0.018
<0 (*n* = 290,862)	1 (reference)	0.73 (0.57–0. 94)	0.60 (0.47–0. 76)	0.51 (0.40–0. 65)	0.46 (0.36–0. 60)	<0.001	
≥0 (*n* = 109,036)	1 (reference)	0.81 (0.61–1. 08)	0.57 (0.43–0.75)	0.49 (0.37–0.64)	0.34 (0.25–0. 46)	<0.001	

^*^Hazard ratios were adjusted for age, sex, ethnicity, household income, educational level, Townsend deprivation index, body mass index, smoking status, alcohol status, Vascular/heart problems, and MET time per week.

TDI, Townsend Deprivation Index; higher scores indicate greater socioeconomic deprivation.

BMI, body mass index; CI, confidence interval.

Subgroup analyses by age, sex, TDI, BMI, educational level, and household income consistently showed that higher healthy sleep scores were associated with a lower risk of incident cervical disc disorders (Supplementary Tables 5–10). Moreover, dose–response relationships were evident across every subgroup of age, sex, TDI, BMI, educational level, and household income, with all *p* values < 0.05. Similarly, a 1-point increase in the healthy sleep score produced consistent effects across subgroup analyses by age, sex, TDI, BMI, educational level, and household income, with all *p* values < 0.05 (Figure 3; Supplementary Tables 5–10).

**Figure 3:**
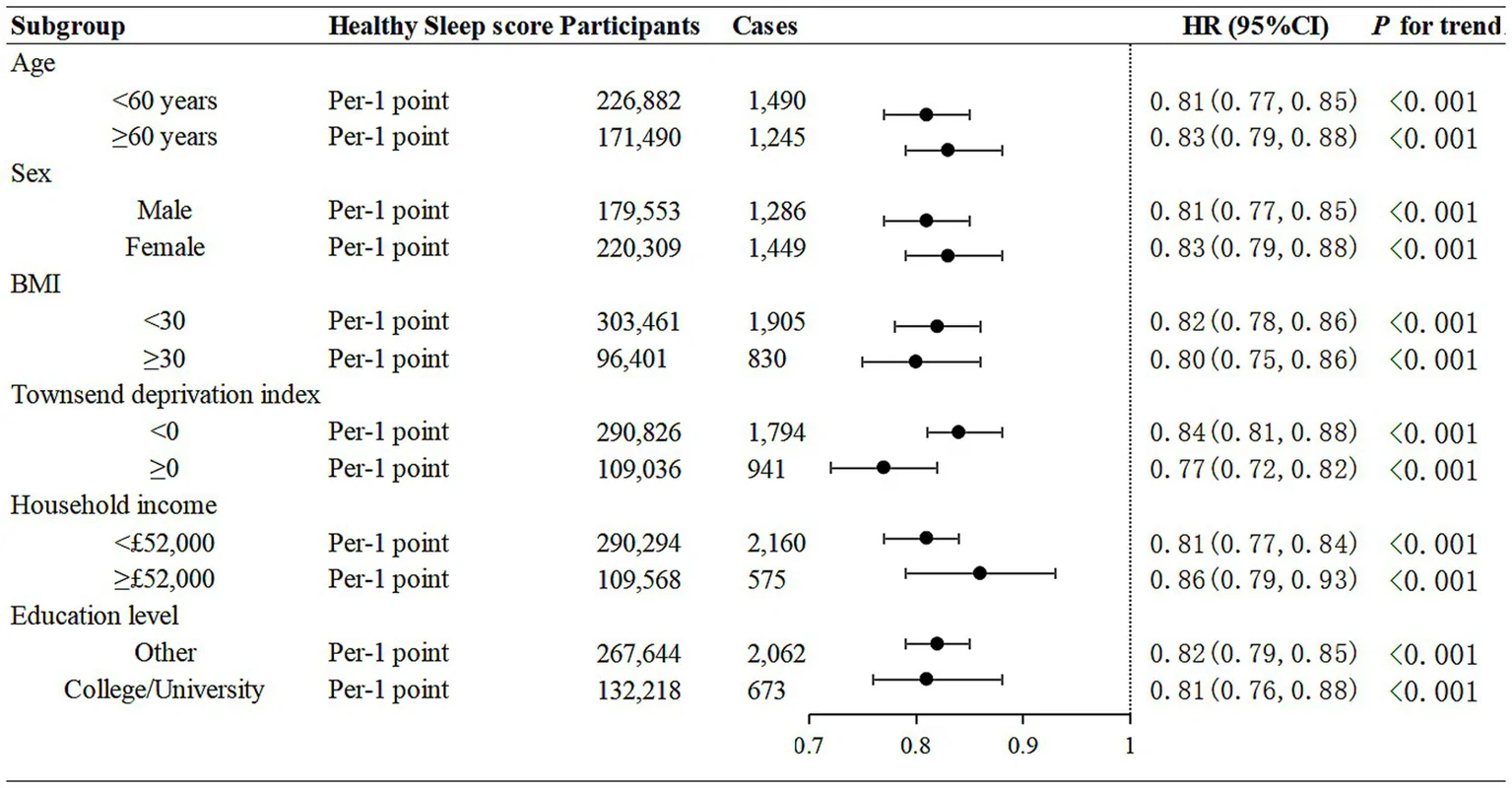
Subgroup analysis of the association between each 1-point increase in healthy sleep score and incident cervical disc disorders. BMI (kg/m^2^), body mass index.

### 3.4 Sensitivity analysis

The association between the healthy sleep score and incident cervical disc disorders remained consistent across sensitivity analyses. First, excluding participants who developed cervical disc disorders within the first 3 or 5 years of follow-up produced similar effect estimates. Second, limiting the sample to white participants while excluding other races did not alter the association. Third, censoring follow-up data on March 11, 2020 (the start of the COVID-19 pandemic) yielded consistent results. Fourth, constructing a weighted healthy sleep score from the five sleep behaviors did not materially change the findings. Fifth, in the covariate adjustment sensitivity analysis, adding M51 and M54 as additional covariates to Model 2 did not materially change the association. Sixth, In the exclusion sensitivity analysis, after removing participants with baseline M51 or M54 diagnoses, the association remained significant. Seventh, after additional adjustment for dorsalgia, depression, and sitting time, the association remained significant (Supplementary Table 11).

## 4 Discussion

In this large prospective cohort of over 390,000 participants, we conducted a comprehensive, systematic assessment of the association between composite healthy sleep patterns and incident cervical disc disorders. To our knowledge, this is among the first prospective cohort studies to examine this association. Over a mean follow-up of 15.83 years, a healthier sleep pattern assessed at baseline was independently associated with a substantially lower risk of developing cervical disc disorders. We also observed a clear dose–response relationship: as the healthy sleep score increased, the risk declined progressively. A similar trend was observed using the weighted sleep score. This association remained robust after adjusting for a wide range of demographic, lifestyle, and socioeconomic factors. It also showed good consistency across multiple prespecified subgroup analyses. These findings provide epidemiological evidence that healthier sleep patterns may be associated with a lower subsequent risk of cervical disc disorders.

Our findings extend previous research in several ways. A large-scale Norwegian study reported that insomnia symptoms predicted chronic musculoskeletal pain over 11 years (Uhlig et al., 2018). A Finnish cohort study found that sleep disorders increased the risk of neck and back pain among firefighters (Lusa et al., 2015). However, these studies focused on pain outcomes rather than disease development. Our study differs by using hospital-recorded diagnoses as the endpoint. It also uses a multidimensional sleep score rather than single sleep indicators. This composite approach captures the holistic nature of sleep health more comprehensively. Subgroup analyses suggested that the inverse association may be stronger among individuals with higher socioeconomic deprivation. This interaction was statistically significant (*p* = 0.018). However, this finding was derived from a secondary analysis and should be interpreted as exploratory. Snoring is a common symptom of obstructive sleep apnea (OSA). However, snoring alone is an imperfect proxy for OSA. OSA involves sleep fragmentation, intermittent hypoxia, and sympathetic activation. These factors may promote systemic inflammation and disc degeneration through pathways beyond those captured by snoring (Tarnoki et al., 2024). The UK Biobank did not include polysomnography or validated OSA screening tools. Therefore, the specific contribution of OSA to cervical disc disorders could not be directly assessed in this study.

The following mechanistic explanations are hypothetical. They are based on previous experimental and clinical studies. They are not directly supported by our data. They are presented to provide context for our observational findings. Several biological pathways may explain the observed association. Sleep is a key regulator of immune function and inflammatory responses. During slow-wave sleep, the body releases growth hormone and suppresses cortisol. This creates a hormonal environment that favors anti-inflammatory activity and tissue repair (Dudek et al., 2017). Unhealthy sleep patterns, in contrast, activate the NF-κB pathway (Ummarino, 2016). This increases the production of pro-inflammatory cytokines, including TNF-α, IL-1β, and IL-6 (Tian et al., 2021; Qiu et al., 2022; Zhang et al., 2019). These cytokines drive intervertebral disc degeneration by inducing matrix metalloproteinases (MMPs). MMPs degrade the disc extracellular matrix and inhibit matrix synthesis. This leads to structural damage and functional loss of the disc. A persistent low-grade inflammatory state also promotes vertebral endplate ossification and neovascularization (Zhang et al., 2019). This further impairs nutrient supply to the disc and creates a vicious cycle. Thus, healthier sleep patterns may be associated with lower risk through effects on systemic inflammation and tissue homeostasis. We emphasize that these mechanistic interpretations are speculative. They are intended to generate hypotheses for future research, not to establish causal pathways. Experimental and clinical studies are needed to validate these mechanisms.

Sleep also influences pain processing. Insufficient sleep lowers pain threshold and tolerance (Chang et al., 2022). This may involve inhibition of the endogenous opioid system and impaired descending pain-inhibitory pathways (Chang et al., 2022). The result is a state of central sensitization (Simpson et al., 2018). In this state, even minor cervical tissue changes can be amplified into clinical symptoms (Finan et al., 2013). In addition, sleep affects daytime behavior. Good sleep quality and abundant energy promote regular physical exercise (Correia et al., 2025). Exercise enhances cervical spine stability and slows degeneration. Conversely, daytime fatigue from insufficient sleep promotes poor posture, such as forward head tilt. This increases biomechanical load on the cervical spine (Tasneem et al., 2026). Fatigue also impairs awareness and raises the risk of accidental neck injury. These behavioral pathways may partly explain the observed association.

Our findings have potential clinical and public health implications. Sleep health is a modifiable behavior. It may serve as a target for preventive interventions. The dose–response relationship suggests that even modest improvements in sleep could be beneficial. The stronger association observed in socioeconomically deprived individuals highlights health equity considerations. Interventions to improve sleep may be particularly valuable in disadvantaged populations. However, our observational design cannot establish causality. Randomized controlled trials are needed to confirm whether sleep improvement reduces the occurrence of cervical disc disorders.

This study has several limitations. Sleep patterns were self-reported at baseline, which may introduce measurement error. Single-time assessment cannot capture changes in sleep over time, potentially leading to non-differential misclassification and biasing estimates toward the null. The predominantly White UK Biobank sample limits generalizability. Outcome ascertainment relied on hospital inpatient records, so mild cases diagnosed in outpatient settings were likely missed, introducing selection bias. Residual confounding from unmeasured factors cannot be excluded, including lumbar spinal stenosis, spondylolisthesis, osteoarthritis, rheumatologic disorders, chronic pain syndromes, occupational physical workload, repetitive mechanical stress, repetitive neck movements, heavy lifting, prolonged computer use, prolonged sedentary work, and obstructive sleep apnea—for which snoring was only a partial proxy. Reverse causation remains possible, as subclinical cervical degeneration may have already existed at baseline and could have affected sleep patterns before clinical diagnosis. We also did not measure biological mediators such as melatonin or inflammatory cytokines, so our mechanistic discussions are speculative and based on prior literature. Finally, our observational design precludes causal inference. These limitations should be considered when interpreting our findings.

This study has several notable strengths. The large sample size provided ample statistical power. The prospective cohort design ensured temporal clarity between exposure and outcome. The long follow-up period of approximately 16 years allowed sufficient time for disease development. The multidimensional sleep score captured the complexity of sleep health. Multiple sensitivity analyses confirmed the robustness of our findings. Finally, the use of ICD-10 diagnoses from hospital records provided objective outcome ascertainment, reducing the risk of reporting bias. These strengths support the validity and generalizability of our findings.

## 5 Conclusion

In this large prospective cohort study, a healthier sleep pattern assessed at baseline was associated with a lower risk of incident cervical disc disorders. The association showed a clear dose–response pattern and remained consistent across multiple sensitivity and subgroup analyses. The association was more pronounced among participants with greater socioeconomic deprivation. These findings suggest that multidimensional sleep health may be relevant to cervical musculoskeletal health. Further studies are warranted to clarify the underlying mechanisms and determine whether improving sleep can reduce the occurrence of cervical disc disorders.

## Data Availability

The original contributions presented in the study are included in the article/Supplementary material, further inquiries can be directed to the corresponding authors.
