# Supplementary material for: Association of healthy sleep patterns with incident cervical disc disorders: a prospective study
Source: Front Neurosci. 2026 Sep 28;20:1940620. doi: 10.3389/fnins.2026.1940620 (PMC13655069; doi:10.3389/fnins.2026.1940620)
Supplement: Supplementary file 1 [file Table_1.DOCX]

SUPPLEMENTARY MATERIALS

**Association of healthy sleep patterns with incident cervical disc disorders: a prospective study**

**Supplementary Table 1. The Assessment of sleep behaviors**

**Supplementary Table 2. Piecewise Cox regression analysis of the association between healthy sleep score and incident cervical disc disorders according to follow-up period**

**Supplementary Table 3. Assessment of the proportional hazards assumption using scaled Schoenfeld residuals before and after model modification**

**Supplementary Table 4. Multivariable-adjusted HRs (95% CIs) for cervical disc disorders by sleep behaviours**

**Supplementary Table 5. Risk of cervical disc disorders associated with healthy sleep score stratified by sex**

**Supplementary Table 6. Risk of cervical disc disorders associated with healthy sleep score stratified by age**

**Supplementary Table 7. Risk of cervical disc disorders associated with healthy sleep score stratified by BMI**

**Supplementary Table 8. Risk of cervical disc disorders associated with healthy sleep score stratified by educational level**

**Supplementary Table 9. Risk of cervical disc disorders associated with healthy sleep score stratified by Household income**

**Supplementary Table 10. Risk of cervical disc disorders associated with healthy sleep score stratified by Townsend deprivation index**

**Supplementary Table 11. Sensitivity analyses regarding risk of incident cervical disc disorders according to healthy sleep score**

**Supplementary Table 1. The Assessment of sleep behaviors**

| **Characteristics** | **UK Biobank Code** | **UK Biobank Questionnaire** | **Healthy sleep(%)** | **Unhealthy sleep (%)** |
| --- | --- | --- | --- | --- |
| **Sleep duration** | 1160 | About how many hours sleep do you get in every 24 hours? (please include naps) | 7-8 h/d | <7 or ≥9 h/d. |
| **Chronotype** | 1180 | Do you consider yourself to be? | Definitely a "morning" person; More a "morning" than "evening" person | More an "evening" than a "morning person; Definitely an "evening" person. |
| **Daytime sleepiness** | 1220 | How likely are you to doze off or fall asleep during the daytime when you don't mean to? (e.g. when working. reading or driving) | Never/rarely/Sometimes | Often/All the Time |
| **Insomnia** | 1200 | Do you have trouble falling asleep at night or do you wake up in the middle of the night? | Never/rarely/Sometimes | Usually |
| **Snoring** | 1210 | Does your partner or a close relative or friend complain about your snoring? | No | Yes |

**Supplementary Table 2. Piecewise Cox regression analysis of the association between healthy sleep score and incident cervical disc disorders according to follow-up period**

| **Variable** | **≤10 years, HR (95% CI)** | ***P* value** | **>10 years, HR (95% CI)** |
| --- | --- | --- | --- |
| Healthy sleep score, per 1-point increase | 0.789 (0.755–0.823) | <0.001 | 0.887 (0.830–0.947) |

Abbreviations: HR, hazard ratio; CI, confidence interval; TDI, Townsend deprivation index; MET, metabolic equivalent of task.

Note: The model was stratified by follow-up period and adjusted for age, sex, ethnicity, educational level, household income, TDI, body mass index, smoking status, alcohol status, vascular/heart problems, and MET time per week. Interaction terms with follow-up period were included for covariates showing evidence of non-proportionality. Follow-up was divided into ≤10 years and >10 years. The interaction term represents the ratio of hazard ratios between the two periods.

**Supplementary Table 3. Assessment of the proportional hazards assumption using scaled Schoenfeld residuals before and after model modification**

| **Covariate** | **Initial Cox model, *P* value** | **Modified piecewise Cox model, *P* value** |
| --- | --- | --- |
| Healthy sleep score | 0.0039 | 0.118 |
| Age | <0.001 | 0.074 |
| Townsend deprivation index | 0.0083 | 0.116 |
| Body mass index | 0.0058 | 0.016 |
| Sex | 0.119 | 0.117 |
| Ethnicity | 0.296 | 0.260 |
| Educational level | 0.521 | 0.874 |
| Household income | 0.336 | 0.683 |
| Smoking status | 0.840 | 0.990 |
| Alcohol status | 0.735 | 0.614 |
| Vascular/heart problems | 0.187 | 0.777 |
| MET time per week | 0.801 | 0.864 |
| Global test | 1.73 × 10⁻⁵ | 0.125 |

Abbreviations: MET, metabolic equivalent of task.
Note: P values were derived from scaled Schoenfeld residual tests. The initial Cox model showed evidence of non-proportionality for healthy sleep score, age, TDI, and BMI. A piecewise Cox model was subsequently fitted by dividing follow-up into ≤10 years and >10 years. Period-specific interaction terms were incorporated for covariates with evidence of time-dependent effects. The modified model was then reassessed using scaled Schoenfeld residuals.

**Supplementary Table 4. Multivariable-adjusted HRs (95% CIs) for cervical disc disorders by sleep behaviours**

| **Sleep behaviours** | **Participants** | **Cases** | **Model 1** | |  | **Model 2** | |  | **Model 3** | |
| --- | --- | --- | --- | --- | --- | --- | --- | --- | --- | --- |
|  |  |  | **HRs (95%CIs)** | ***P*** |  | **HRs (95%CIs)** | ***P*** |  | **HRs (95%CIs)** | ***P*** |
| Sleep duration |  |  |  |  |  |  |  |  |  |  |
| Normal (7-8 h) | 273294 | 1593 | 1 (reference) | - |  | 1 (reference) | - |  | 1 (reference) | - |
| Short (<7 h) | 95994 | 898 | 1.62(1.49,1.76) | <0.001 |  | 1.52(1.40,1.65) | <0.001 |  | 1.41(1.30,1.53) | <0.001 |
| Long (>8 h) | 30574 | 244 | 1.41(1.23,1.61) | <0.001 |  | 1.27(1.11,1.46) | <0.001 |  | 1.19(1.04,1.36) | 0.014 |
| Chronotype |  |  |  |  |  |  |  |  |  |  |
| Morningness | 107714 | 745 | 1 (reference) | - |  | 1 (reference) | - |  | 1 (reference) | - |
| Morningness than eveningness | 143003 | 893 | 0.90 (0.81-0.99) | 0.030 |  | 0.94 (0.85-1.04) | 0.229 |  | 0.96(0.87,1.06) | 0.424 |
| Eveningness than morningness | 113901 | 818 | 1.04 (0.94-1.14) | 0.478 |  | 1.07 (0.97-1.18) | 0.187 |  | 1.05(0.95,1.16) | 0.321 |
| Eveningness | 35244 | 279 | 1.15 (1.00-1.32) | 0.049 |  | 1.19 (1.04-1.37) | 0.013 |  | 1.12(0.97,1.28) | 0.125 |
| Daytime sleepiness |  |  |  |  |  |  |  |  |  |  |
| Never/rarely | 305277 | 1946 | 1 (reference) | - |  | 1 (reference) | - |  | 1 (reference) | - |
| Sometimes | 83802 | 665 | 1.26 (1.16-1.38) | <0.001 |  | 1.17 (1.07-1.28) | <0.001 |  | 1.12(1.02,1.23) | 0.013 |
| Always/Often | 10783 | 124 | 1.86 (1.55-2.23) | <0.001 |  | 1.65 (1.37-1.98) | <0.001 |  | 1.44(1.20,1.73) | <0.001 |
| Insomnia |  |  |  |  |  |  |  |  |  |  |
| Never/rarely | 97676 | 475 | 1 (reference) | - |  | 1 (reference) | - |  | 1 (reference) | - |
| Sometimes | 191260 | 1214 | 1.31 (1.18-1.46) | <0.001 |  | 1.29 (1.16-1.43) | <0.001 |  | 1.25(1.12,1.39) | <0.001 |
| Usually | 110926 | 1046 | 1.96 (1.76-2.19) | <0.001 |  | 1.88 (1.68-2.10) | <0.001 |  | 1.68(1.50,1.88) | <0.001 |
| Snoring |  |  |  |  |  |  |  |  |  |  |
| No | 251217 | 1633 | 1 (reference) | - |  | 1 (reference) | - |  | 1 (reference) | - |
| Yes | 148645 | 1102 | 1.14 (1.06-1.23) | <0.001 |  | 1.12 (1.03-1.21) | 0.006 |  | 1.03(0.95,1.11) | 0.501 |

A Cox proportional hazard model was used. Model 1: unadjusted  model; Model 2, age, sex, ethnicity, household income,educational level, and Townsend deprivation index were adjusted for in this model; Model 3, body mass index, smoking status, alcohol status, vascular/heart problems, MET time per week, dorsalgia, depression, and time of sitting additionally were adjusted for in this model.

Sleep behaviors were measured at baseline and do not reflect changes during follow‑up.

HR, hazard ratio; CI, confidence interval.

**Supplementary Table 5. Risk of cervical disc disorders associated with healthy sleep score stratified by sex**

| **Sex subgroup** | **Healthy sleep score** | | | | | **Per 1-point increment** | ***P* for trend** |
| --- | --- | --- | --- | --- | --- | --- | --- |
|  | **0-1** | **2** | **3** | **4** | **5** |  |  |
| **Male (N=179553)** |  |  |  |  |  |  |  |
| Participants, n | 4704 | 21282 | 54325 | 67617 | 31625 | **-** | **-** |
| Incident cervical disc disorders, n | 72 | 232 | 405 | 409 | 168 | **-** | **-** |
| Hazard ratio for incident cervical disc disorders (95% CI) | |  |  |  |  |  |  |
| Crude model | Reference | 0.70(0.54,0.92) | 0.48(0.37,0.61) | 0.39(0.30,0.49) | 0.34(0.26,0.44) | 0.76(0.73,0.81) | <0.001 |
| Adjusted model 1 | Reference | 0.74(0.57,0.96) | 0.52(0.40,0.67) | 0.43(0.33,0.55) | 0.38(0.29,0.50) | 0.79(0.75,0.83) | <0.001 |
| Adjusted model 2 | Reference | 0.80(0.61,1.04) | 0.59(0.46,0.76) | 0.51(0.39,0.66) | 0.46(0.35,0.62) | 0.82(0.78,0.87) | <0.001 |
| **Female (N=220309)** |  |  |  |  |  |  |  |
| Participants, n | 4937 | 23645 | 58125 | 79541 | 54061 | - | - |
| Incident cervical disc disorders, n | 68 | 228 | 430 | 477 | 246 | - | - |
| Hazard ratio for incident cervical disc disorders (95% CI) | |  |  |  |  |  |  |
| Crude model | Reference | 0.69(0.53,0.91) | 0.53(0.41,0.68) | 0.43(0.33,0.55) | 0.32(0.25,0.42) | 0.77(0.74,0.81) | <0.001 |
| Adjusted model 1 | Reference | 0.72(0.55,0.95) | 0.57(0.44,0.74) | 0.48(0.37,0.61) | 0.37(0.28,0.48) | 0.80(0.76,0.83) | <0.001 |
| Adjusted model 2 | Reference | 0.80(0.61,1.06) | 0.68(0.53,0.88) | 0.61(0.47,0.79) | 0.49(0.37,0.65) | 0.85(0.81,0.89) | <0.001 |

A Cox proportional hazard model was used. Adjusted model 1, age, ethnicity, household income,educational level, and Townsend deprivation index were adjusted for in this model; adjusted model 2, body mass index, smoking status, alcohol status, vascular/heart problems, MET time per week, dorsalgia, depression, and time of sitting additionally were adjusted for in this model.

Sleep behaviors were measured at baseline and do not reflect changes during follow‑up.

CI, confidence interval.

**Supplementary Table 6. Risk of cervical disc disorders associated with healthy sleep score stratified by age**

| **Age subgroup** | **Healthy sleep score** | | | | | **Per 1-point increment** | ***P* for trend** |
| --- | --- | --- | --- | --- | --- | --- | --- |
|  | **0-1** | **2** | **3** | **4** | **5** |  |  |
| **<60 years (N=228372)** |  |  |  |  |  |  |  |
| Participants, n | 5630 | 25564 | 63331 | 84210 | 49637 | **-** | **-** |
| Incident cervical disc disorders, n | 89 | 270 | 427 | 484 | 220 | **-** | **-** |
| Hazard ratio for incident cervical disc disorders (95% CI) | |  |  |  |  |  |  |
| Crude model | Reference | 0.66(0.52,0.84) | 0.42(0.33,0.53) | 0.36(0.28,0.45) | 0.27(0.21,0.35) | 0.74(0.71,0.78) | <0.001 |
| Adjusted model 1 | Reference | 0.70(0.55,0.90) | 0.47(0.37,0.59) | 0.41(0.33,0.52) | 0.33(0.25,0.42) | 0.77(0.74,0.81) | <0.001 |
| Adjusted model 2 | Reference | 0.79(0.62,1.01) | 0.57(0.45,0.72) | 0.54(0.43,0.68) | 0.45(0.35,0.58) | 0.83(0.79,0.87) | <0.001 |
| **≥60 years (N=171490)** |  |  |  |  |  |  |  |
| Participants, n | 4011 | 19363 | 49119 | 62948 | 36049 | - | - |
| Incident cervical spondylosis, n | 51 | 190 | 408 | 402 | 194 | - | - |
| Hazard ratio for incident cervical disc disorders (95% CI) | |  |  |  |  |  |  |
| Crude model | Reference | 0.76(0.56,1.04) | 0.64(0.48,0.86) | 0.49(0.36,0.65) | 0.41(0.30,0.56) | 0.80(0.76,0.84) | <0.001 |
| Adjusted model 1 | Reference | 0.79(0.58,1.07) | 0.67(0.50,0.90) | 0.52(0.39,0.70) | 0.45(0.33,0.61) | 0.82(0.77,0.86) | <0.001 |
| Adjusted model 2 | Reference | 0.84(0.61,1.14) | 0.75(0.56,1.00) | 0.60(0.45,0.81) | 0.53(0.38,0.72) | 0.85(0.80,0.89) | <0.001 |

A Cox proportional hazard model was used. Adjusted model 1, sex, ethnicity, household income,educational level, and Townsend deprivation index were adjusted for in this model; adjusted model 2, body mass index, smoking status, alcohol status, vascular/heart problems, MET time per week, dorsalgia, depression, and time of sitting additionally were adjusted for in this model.

Sleep behaviors were measured at baseline and do not reflect changes during follow‑up.

CI, confidence interval.

**Supplementary Table 7. Risk of cervical disc disorders associated with healthy sleep score stratified by BMI**

| **BMI subgroup** | **Healthy sleep score** | | | | | **Per 1-point increment** | ***P* for trend** |
| --- | --- | --- | --- | --- | --- | --- | --- |
|  | **0-1** | **2** | **3** | **4** | **5** |  |  |
| **BMI<30 kg/m^2^ (N=303461)** |  |  |  |  |  |  |  |
| Participants, n | 5466 | 29624 | 81710 | 114911 | 71750 | **-** | **-** |
| Incident cervical disc disorders, n | 74 | 274 | 573 | 652 | 332 | **-** | **-** |
| Hazard ratio for incident cervical disc disorders (95% CI) | |  |  |  |  |  |  |
| Crude model | Reference | 0.67(0.52,0.87) | 0.51(0.40,0.65) | 0.41(0.32,0.52) | 0.33(0.26,0.43) | 0.78(0.75,0.82) | <0.001 |
| Adjusted model 1 | Reference | 0.70(0.54,0.91) | 0.54(0.43,0.69) | 0.45(0.36,0.58) | 0.38(0.29,0.49) | 0.80(0.77,0.84) | <0.001 |
| Adjusted model 2 | Reference | 0.75(0.58,0.97) | 0.61(0.48,0.78) | 0.53(0.42,0.68) | 0.46(0.35,0.59) | 0.84(0.81,0.88) | <0.001 |
| **BMI≥30 kg/m^2^ (N=96401)** |  |  |  |  |  |  |  |
| Participants, n | 4175 | 15303 | 30740 | 32247 | 13936 | - | - |
| Incident cervical disc disorders, n | 66 | 186 | 262 | 234 | 82 | - | - |
| Hazard ratio for incident cervical disc disorders (95% CI) | |  |  |  |  |  |  |
| Crude model | Reference | 0.76(0.58,1.01) | 0.53(0.41,0.70) | 0.45(0.34,0.59) | 0.36(0.26,0.50) | 0.78(0.73,0.83) | <0.001 |
| Adjusted model 1 | Reference | 0.79(0.60,1.05) | 0.57(0.43,0.75) | 0.49(0.37,0.64) | 0.40(0.29,0.55) | 0.79(0.74,0.84) | <0.001 |
| Adjusted model 2 | Reference | 0.85(0.64,1.13) | 0.63(0.48,0.83) | 0.56(0.43,0.74) | 0.47(0.34,0.65) | 0.82(0.77,0.88) | <0.001 |

A Cox proportional hazard model was used. Adjusted model 1, age, sex, ethnicity, household income,educational level, and Townsend deprivation index were adjusted for in this model; adjusted model 2, smoking status, alcohol status, vascular/heart problems, MET time per week, dorsalgia, depression, and time of sitting additionally were adjusted for in this model.

Sleep behaviors were measured at baseline and do not reflect changes during follow‑up.

CI, confidence interval.

**Supplementary Table 8. Risk of cervical disc disorders associated with healthy sleep score stratified by educational level**

| **Education subgroup** | **Healthy sleep score** | | | | | **Per 1-point increment** | ***P* for trend** |
| --- | --- | --- | --- | --- | --- | --- | --- |
|  | **0-1** | **2** | **3** | **4** | **5** |  |  |
| **College/University (N=132218)** |  |  |  |  |  |  |  |
| Participants, n | 2371 | 12412 | 34902 | 50933 | 31600 | **-** | **-** |
| Incident cervical disc disorders, n | 30 | 97 | 185 | 245 | 116 | **-** | **-** |
| Hazard ratio for incident cervical disc disorders (95% CI) | |  |  |  |  |  |  |
| Crude model | Reference | 0.61(0.41,0.92) | 0.41(0.28,0.61) | 0.37(0.26,0.55) | 0.28(0.19,0.42) | 0.77(0.72,0.83) | <0.001 |
| Adjusted model 1 | Reference | 0.63(0.42,0.94) | 0.43(0.29,0.64) | 0.40(0.27,0.58) | 0.31(0.21,0.46) | 0.79(0.73,0.85) | <0.001 |
| Adjusted model 2 | Reference | 0.69(0.45,1.03) | 0.51(0.34,0.75) | 0.49(0.33,0.73) | 0.40(0.26,0.60) | 0.84(0.78,0.90) | <0.001 |
| **Other (N=267644)** |  |  |  |  |  |  |  |
| Participants, n | 7270 | 32515 | 77548 | 96225 | 54086 | - | - |
| Incident cervical disc disorders, n | 110 | 363 | 650 | 641 | 298 | - | - |
| Hazard ratio for incident cervical disc disorders (95% CI) | |  |  |  |  |  |  |
| Crude model | Reference | 0.73(0.59,0.90) | 0.54(0.44,0.67) | 0.43(0.35,0.53) | 0.35(0.28,0.44) | 0.78(0.75,0.81) | <0.001 |
| Adjusted model 1 | Reference | 0.76(0.61,0.94) | 0.58(0.47,0.71) | 0.47(0.38,0.57) | 0.39(0.31,0.49) | 0.79(0.76,0.83) | <0.001 |
| Adjusted model 2 | Reference | 0.83(0.67,1.03) | 0.67(0.55,0.82) | 0.57(0.46,0.70) | 0.50(0.40,0.62) | 0.84(0.80,0.87) | <0.001 |

A Cox proportional hazard model was used. Adjusted model 1, age,sex, ethnicity, household income, and Townsend deprivation index were adjusted for in this model; adjusted model 2, body mass index, smoking status, alcohol status, vascular/heart problems, MET time per week, dorsalgia, depression, and time of sitting additionally were adjusted for in this model.

Sleep behaviors were measured at baseline and do not reflect changes during follow‑up.

CI, confidence interval.

**Supplementary Table 9. Risk of cervical disc disorders associated with healthy sleep score stratified by household income**

| **Household income subgroup** | **Healthy sleep score** | | | | | **Per 1-point increment** | ***P* for trend** |
| --- | --- | --- | --- | --- | --- | --- | --- |
|  | **0-1** | **2** | **3** | **4** | **5** |  |  |
| **Household income <£52,000 (N=290294)** |  |  |  |  |  |  |  |
| Participants, n | 7630 | 34361 | 82956 | 104983 | 60364 | **-** | **-** |
| Incident cervical disc disorders, n | 116 | 387 | 664 | 680 | 313 | **-** | **-** |
| Hazard ratio for incident cervical disc disorders (95% CI) | |  |  |  |  |  |  |
| Crude model | Reference | 0.73(0.60,0.90) | 0.52(0.42,0.63) | 0.42(0.34,0.51) | 0.33(0.27,0.41) | 0.76(0.73,0.79) | <0.001 |
| Adjusted model 1 | Reference | 0.76(0.62,0.93) | 0.55(0.45,0.66) | 0.45(0.37,0.55) | 0.36(0.29,0.45) | 0.78(0.75,0.81) | <0.001 |
| Adjusted model 2 | Reference | 0.83(0.68,1.03) | 0.64(0.53,0.78) | 0.55(0.45,0.68) | 0.47(0.38,0.59) | 0.83(0.80,0.86) | <0.001 |
| **Household income ≥£52,000 (N=109568)** |  |  |  |  |  |  |  |
| Participants, n | 2011 | 10566 | 29494 | 42175 | 25322 | - | - |
| Incident cervical disc disorders, n | 24 | 76 | 171 | 206 | 101 | - | - |
| Hazard ratio for incident cervical disc disorders (95% CI) | |  |  |  |  |  |  |
| Crude model | Reference | 0.57(0.36,0.91) | 0.48(0.31,0.73) | 0.40(0.26,0.61) | 0.33(0.21,0.51) | 0.81(0.74,0.87) | <0.001 |
| Adjusted model 1 | Reference | 0.59(0.37,0.94) | 0.51(0.33,0.78) | 0.44(0.29,0.67) | 0.37(0.24,0.57) | 0.83(0.77,0.90) | <0.001 |
| Adjusted model 2 | Reference | 0.65(0.41,1.04) | 0.59(0.38,0.91) | 0.54(0.35,0.83) | 0.47(0.30,0.74) | 0.87(0.81,0.95) | 0.001 |

A Cox proportional hazard model was used. Adjusted model 1, age,sex, ethnicity, educational level, and Townsend deprivation index were adjusted for in this model; adjusted model 2, body mass index, smoking status, alcohol status, vascular/heart problems, MET time per week, dorsalgia, depression, and time of sitting additionally were adjusted for in this model.

Sleep behaviors were measured at baseline and do not reflect changes during follow‑up.

CI, confidence interval.

**Supplementary Table 10. Risk of cervical disc disorders associated with healthy sleep score stratified by Townsend deprivation index**

| **Townsend deprivation index subgroup** | **Healthy sleep score** | | | | | **Per 1-point increment** | ***P* for trend** |
| --- | --- | --- | --- | --- | --- | --- | --- |
|  | **0-1** | **2** | **3** | **4** | **5** |  |  |
| **Townsend deprivation index <0 (N=290826)** |  |  |  |  |  |  |  |
| Participants, n | 6063 | 30651 | 80732 | 108709 | 64671 | **-** | **-** |
| Incident cervical disc disorders, n | 76 | 266 | 545 | 599 | 308 | **-** | **-** |
| Hazard ratio for incident cervical disc disorders (95% CI) | |  |  |  |  |  |  |
| Crude model | Reference | 0.69(0.53,0.88) | 0.53(0.42,0.67) | 0.43(0.34,0.55) | 0.37(0.29,0.48) | 0.80(0.77,0.84) | <0.001 |
| Adjusted model 1 | Reference | 0.69(0.54,0.90) | 0.54(0.43,0.69) | 0.45(0.35,0.57) | 0.39(0.31,0.51) | 0.81(0.78,0.85) | <0.001 |
| Adjusted model 2 | Reference | 0.76(0.59,0.98) | 0.63(0.50,0.81) | 0.55(0.44,0.71) | 0.51(0.40,0.66) | 0.87(0.83,0.91) | <0.001 |
| **Townsend deprivation index ≥0 (N=109036)** |  |  |  |  |  |  |  |
| Participants, n | 3578 | 14276 | 31718 | 38449 | 21015 | - | - |
| Incident cervical disc disorders, n | 64 | 194 | 290 | 287 | 106 | - | - |
| Hazard ratio for incident cervical disc disorders (95% CI) | |  |  |  |  |  |  |
| Crude model | Reference | 0.75(0.57,1.00) | 0.50(0.38,0.66) | 0.41(0.31,0.53) | 0.27(0.20,0.37) | 0.73(0.69,0.77) | <0.001 |
| Adjusted model 1 | Reference | 0.78(0.59,1.04) | 0.54(0.41,0.70) | 0.45(0.34,0.59) | 0.31(0.22,0.42) | 0.75(0.71,0.79) | <0.001 |
| Adjusted model 2 | Reference | 0.85(0.64,1.13) | 0.62(0.47,0.81) | 0.54(0.41,0.71) | 0.38(0.27,0.52) | 0.79(0.74,0.84) | <0.001 |

A Cox proportional hazard model was used. Adjusted model 1, age,sex, ethnicity, and household income,educational level were adjusted for in this model; adjusted model 2, body mass index, smoking status, alcohol status, vascular/heart problems, MET time per week, dorsalgia, depression, and time of sitting additionally were adjusted for in this model.

Sleep behaviors were measured at baseline and do not reflect changes during follow‑up.

CI, confidence interval.

**Supplementary Table 11. Sensitivity analyses regarding risk of incident cervical disc disorders according to healthy sleep score**

| **Healthy sleep score** | **Participants, n** | **Cervical disc disorders, n** | **Adjusted HR (95% CI)** | | ***P* value** | | ***P* for trend** |
| --- | --- | --- | --- | --- | --- | --- | --- |
| **Sensitivity analysis 1: excluding cervical disc disorders participants diagnosed within 3 years after baseline (N= 396089)** | | | | | | | |
| Per 1-point increment | **-** | **-** | 0.85(0.82,0.89) | | <0.001 | | <0.001 |
| 0-1 | 9491 | 110 | Reference | | - | |  |
| 2 | 44338 | 363 | 0.81(0.65,1.00) | | 0.048 | |  |
| 3 | 111325 | 682 | 0.66(0.54,0.81) | | <0.001 | |  |
| 4 | 145879 | 733 | 0.58(0.48,0.72) | | <0.001 | |  |
| 5 | 85056 | 345 | 0.50(0.40,0.63) | | <0.001 | |  |
| **Sensitivity analysis 2: excluding cervical disc disorders participants diagnosed within 5 years after baseline (N=392408)** | | | | | | | |
| Per 1-point increment | **-** | **-** | 0.86(0.82,0.90) | | <0.001 | | <0.001 |
| 0-1 | 9339 | 85 | Reference | | - | |  |
| 2 | 43808 | 293 | 0.84(0.66,1.06) | | 0.145 | |  |
| 3 | 110223 | 569 | 0.70(0.56,0.88) | | 0.003 | |  |
| 4 | 144645 | 611 | 0.62(0.49,0.78) | | <0.001 | |  |
| 5 | 84393 | 284 | 0.53(0.41,0.67) | | <0.001 | |  |
| **Sensitivity analysis 3: Retain only white people, exclude individuals of other races. (N=380746)** | | | | | | | |
| Per 1-point increment | - | - | 0.84(0.81,0.87) | | <0.001 | | <0.001 |
| 0-1 | 8991 | 132 | Reference | | - | |  |
| 2 | 42576 | 435 | 0.79(0.65,0.96) | | 0.018 | |  |
| 3 | 106979 | 779 | 0.61(0.51,0.74) | | <0.001 | |  |
| 4 | 140288 | 843 | 0.55(0.45,0.66) | | <0.001 | |  |
| 5 | 81912 | 393 | 0.47(0.38,0.57) | | <0.001 | |  |
| **Sensitivity analysis 4: before March 11, 2020 (the start date of the COVID-19 pandemic) (N= 399862)** | | | | | | | |
| Per 1-point increment | - | - | 0.81(0.78,0.85) | | <0.001 | | <0.001 |
| 0-1 | 9641 | 116 | Reference | | - | |  |
| 2 | 44927 | 367 | 0.77(0.63,0.95) | | 0.016 | |  |
| 3 | 112450 | 650 | 0.60(0.49,0.73) | | <0.001 | |  |
| 4 | 147158 | 653 | 0.49(0.40,0.60) | | <0.001 | |  |
| 5 | 85686 | 311 | 0.43(0.34,0.53) | | <0.001 | |  |
| **Sensitivity analysis 5: by weighted healthy sleep score in UK Biobank (N=399862)** | | | | | | | |
| Per 1-point increment | - | - | 0.81(0.78,0.86) | | <0.001 | | <0.001 |
| 0-1 | 9641 | 140 | Reference | | - | |  |
| 1-2 | 44927 | 460 | 0.80(0.67,0.97) | | 0.025 | |  |
| 2-3 | 210604 | 1382 | 0.59(0.49,0.70) | | <0.001 | |  |
| 3-5 | 134690 | 753 | 0.54(0.45,0.64) | | <0.001 | |  |
| **Sensitivity analysis 6: Cox regression analyses were additionally adjusted for M51 (other intervertebral disc disorders) and M54 (dorsalgia, including neck pain) (N=399862)** | | | | | | | |
| Per 1-point increment | - | - | 0.79(0.76,0.82) | | <0.001 | | <0.001 |
| 0-1 | 9641 | 140 | Reference | | - | |  |
| 2 | 44927 | 460 | 0.81(0.67,0.98) | | 0.030 | |  |
| 3 | 112450 | 835 | 0.64(0.54,0.77) | | <0.001 | |  |
| 4 | 147158 | 886 | 0.56(0.47,0.67) | | <0.001 | |  |
| 5 | 85686 | 414 | 0.48(0.39,0.58) | | <0.001 | |  |
| **Sensitivity analysis 7: We excluded all participants with a baseline diagnosis of M51 (other intervertebral disc disorders) and M54 (dorsalgia, including neck pain) and re‑ran the Cox regression analysis (N=352963)** | | | | | | | |
| Per 1-point increment | - | - | 0.84(0.80,0.87) | | <0.001 | | <0.001 |
| 0-1 | 7952 | 99 | Reference | | - | |  |
| 2 | 38319 | 300 | 0.68(0.54,0.85) | | <0.001 | |  |
| 3 | 98266 | 579 | 0.54(0.43,0.67) | | <0.001 | |  |
| 4 | 131196 | 658 | 0.48(0.39,0.60) | | <0.001 | |  |
| 5 | 77230 | 318 | 0.41(0.33,0.52) | | <0.001 | |  |
| **Sensitivity analysis 8: cox regression analyses were additionally adjusted for dorsalgia, depression, and sitting time (N=399862)** | | | | | | | |
| Per 1-point increment | - | - | | 0.84(0.81,0.87) | | <0.001 | <0.001 |
| 0-1 | 9641 | 140 | | Reference | | - |  |
| 2 | 44927 | 460 | | 0.80(0.66,0.97) | | <0.001 |  |
| 3 | 112450 | 835 | | 0.63(0.53,0.76) | | <0.001 |  |
| 4 | 147158 | 886 | | 0.56(0.46,0.67) | | <0.001 |  |
| 5 | 85686 | 414 | | 0.47(0.39,0.58) | | <0.001 |  |

A Cox proportional hazard model was used. All hazard ratios were calculated by adjusting for the following covariates: age, sex, ethnicity, household income, educational level, Townsend deprivation index, body mass index, smoking status, alcohol status, vascular/heart problems, MET time per week, dorsalgia, depression, and time of sitting.

Sleep behaviors were measured at baseline and do not reflect changes during follow‑up.

HR, hazard ratio; CI, confidence interval
